# Dentin matrix degradation by host matrix metalloproteinases: inhibition and clinical perspectives toward regeneration

**DOI:** 10.3389/fphys.2013.00308

**Published:** 2013-11-01

**Authors:** Catherine Chaussain, Tchilalo Boukpessi, Mayssam Khaddam, Leo Tjaderhane, Anne George, Suzanne Menashi

**Affiliations:** ^1^EA 2496 Dental School University Paris DescartesMontrouge, France; ^2^Odontology Departments (Bretonneau and Charles Foix), AP-HPParis, France; ^3^Institute of Dentistry, University of TurkuTurku, Finland; ^4^Institute of Dentistry, University of Oulu, Oulu University HospitalOulu, Finland; ^5^Department of Oral Biology, University of IllinoisChicago, IL, USA; ^6^Laboratoire CRRET, Université Paris-Est, CNRSCréteil, France

**Keywords:** dentin, carious process, matrix degradation, MMPs, SIBLINGs, MMP inhibitors, biopeptides, regeneration

## Abstract

Bacterial enzymes have long been considered solely accountable for the degradation of the dentin matrix during the carious process. However, the emerging literature suggests that host-derived enzymes, and in particular the matrix metalloproteinases (MMPs) contained in dentin and saliva can play a major role in this process by their ability to degrade the dentin matrix from within. These findings are important since they open new therapeutic options for caries prevention and treatment. The possibility of using MMP inhibitors to interfere with dentin caries progression is discussed. Furthermore, the potential release of bioactive peptides by the enzymatic cleavage of dentin matrix proteins by MMPs during the carious process is discussed. These peptides, once identified, may constitute promising therapeutical tools for tooth and bone regeneration.

## Introduction

Enamel is the hardest structure of the organism, containing about 96% of mineral but only traces of organic matrix (less than 1%). The enamel carious process involves physicochemical reactions, where the mineral is dissolved by acids liberated by cariogenic bacteria. In contrast, the dentin is less mineralized and contains an abundant organic material (19–20%), mainly composed of type I collagen. Therefore, the dentin carious process strongly differs from the enamel carious process. First, the bacterial acids dissolve the dentin mineral, which progressively uncovers the organic dentin extracellular matrix (ECM). Second, proteases degrade the ECM components allowing the progression of cariogenic bacteria toward pulp tissues. Such progression is facilitated by the tubular nature of the circumpulpal dentin. It has long been assumed that dentin organic matrix is degraded by proteases secreted by cariogenic bacteria. However, bacterial collagenases have been shown to be highly pH sensitive and not able to resist the acidic fall (pH 4.3) during the demineralization phase of a pH cycling model (Kawasaki and Featherstone, 1997), suggesting that this enzyme's contribution to dentin matrix degradation may be limited. Therefore, the potential role of host-derived proteases and in particular MMPs, in dentin matrix degradation has been introduced. MMPs form a mammalian family of ECM proteinases involved in normal and pathological events in almost all tissues of the organism including the tooth. MMPs present specific properties and characteristics (Brinckerhoff and Matrisian, 2002), including their ability to cleave matrix components, their dependence on a zinc ion for activity, the requirement that the enzymes be activated by the cleavage of a prodomain, the conservation of specific amino acid sequences between family members, and inhibition of their enzymatic activity by endogenous tissue inhibitors of metalloproteinases (TIMPs) (Visse and Nagase, 2003). Numerous studies have demonstrated endogenous proteolytic enzyme activity within the dentin, including matrix metalloproteinases MMP-2, MMP-9 and MMP-3 (Tjaderhane et al., 1998; Mazzoni et al., 2007) which were shown to be required for normal dentin formation. Once the neoformed dentin matrix mineralizes, some of these enzymes remain trapped in the calcified matrix, either under active or proenzyme forms (Palosaari et al., 2003). It can be hypothesized that the cariogenic demineralization process not only re-exposes these enzymes but also potentially induces their activation. In addition, the saliva, which bathes the dentin carious lesion, contains several proteases that can also participate in the organic matrix degradation (Tjaderhane et al., 1998; Van Strijp et al., 2003). The aim of the present article is to review the role of the host metalloproteinases (MMPs) in the dentin carious process. The ability of MMPs to liberate bioactive peptides and degrade dentin matrix proteins as well as the potential use of protease inhibitors to block their activity in the treatment of caries will be discussed.

## Dentin matrix proteins

Dentin ECM consists of a 3D scaffold that is mainly formed of type I collagen fibrils (90%). Types III and V collagens were also identified in this scaffold but at lower levels (1–3%) (Goldberg and Smith, 2004; Opsahl Vital et al., 2012). Thin collagen fibrils are secreted at the apical pole of the odontoblast body to form the predentin (which is equivalent to the osteoid matrix in bone). Collagen fibrils undergo fibrillogenesis along the predentin by processes of self-assembly and cross-linking to form a template which can be efficiently mineralized. Non-collagenous proteins (NCPs), which have been the focus of intense studies in the last decade because of their potential roles in the regulation of bone and dentin mineralization, constitute the remaining 10% of the ECM scaffold. It has been suggested that some NCPs are associated with specific sites on collagen fibrils to regulate the nucleation and growth of hydroxyapatite (HAP) crystals. For example, the glycoaminoglycans (GAG) part of the proteoglycans (PG) was shown to be important for the collagen fibril maturation by delaying apatite crystals deposition to allow the fibrils to reach a diameter sufficient to achieve proper mineralization (Embery et al., 2001). The importance of NCPs in the mineralization process has been well demonstrated by mutation studies and by experimental suppression of NCP genes. These experiments have highlighted the importance in the mineralization process of SIBLINGs (Small Integrin Binding LIgand N-linked Glycoproteins), a phosphoprotein family in which mutations are associated with abnormal phenotypes in the mineralization of bone and/or dentin (Qin et al., 2004; Opsahl Vital et al., 2012). This family includes dentin sialophosphoprotein (DSPP), dentin matrix protein 1 (DMP1), bone sialoprotein (BSP), matrix extracellular phosphorylated glycoprotein (MEPE), and osteopontin (OPN). It appears that the associations of these highly phosphorylated and acidic NCPs with specific sites on collagen molecules are essential in promoting the nucleation and growth of apatite crystals. All SIBLINGs were identified in dentin and bone ECM, but a high rate of DSPP expression was shown to be specific to dentin. The SIBLING members carry an arginine–glycine–aspartate (RGD) cell adhesion domain and a highly conserved acidic serine and aspartate-rich motif (ASARM) (Rowe et al., 2000; Fisher and Fedarko, 2003). Noteworthy, the function of ASARM domain in bone and teeth mineralization (apatite crystals nucleator or inhibitor) is at present debated by the scientific community, in particular its implication in pathological processes such as inherited rickets (Addison and Mckee, 2010; David and Quarles, 2010; Rowe, 2012). It is of interest that, in addition to binding integrins SIBLINGs, may also specifically bind and activate several MMPs in the ECM suggesting that they could be involved in dentin matrix degradation (Fedarko et al., 2004).

Dentin contains non-phosphorylated proteins, such as osteonectin (SPARC protein or BM40) and proteins with gamma-carboxylated glutamates (Gla) residues (osteocalcin and matrix Gla protein-MGP-). While osteonectin may contribute to the mineralization process, osteocalcin and MGP have been suggested to regulate HAP crystal nucleation (Bronckers et al., 1998; Onishi et al., 2005; Kaipatur et al., 2008). The small leucine-rich proteoglycans (SLRPs), such as decorin, biglycan, fibromodulin, lumican, and osteoadherin, have also been identified in predentin and dentin (Goldberg et al., 1987, 2003a). Although not specific to dentin and can be found in other mineralized or non-mineralized tissues, they have been implicated in dentin formation and mineralization (Embery et al., 2001). They are thought to be involved in the transport of collagen fibrils through the predentin and in collagen fibrillogenesis (Goldberg et al., 2003a). Predentin is also rich in dermatan and chondroitin sulphate-containing (PG). It is of interest that adjacent to the mineralization front, predentin contains a large quantity of keratan sulphate-containing PG associated with a dramatic decrease in dermatan and chondroitin sulphate-containing PG. This switch in the proteoglycan type was attributed to MMP-3, which is closely related to a control of the dentin mineralization process (Hall et al., 1999).

Dentin also contains α-2-Heremans Schmid-glycoprotein (alpha-2-HSglycoprotein, AHSG), a serum protein currently known as Fetuin-A. Fetuin-A is a serum protein produced in the liver that concentrates in mineralized tissues, especially dentin because of its high affinity for hydroxyapatite (Mazzoni et al., 2012). The role for fetuins is to inhibit undesirable ectopic calcification without affecting normal bone or dentin mineralization. Interestingly, Fetuin-A can differentially control MMP-2 and -9 activities, both as an inhibitor, activator or stabilizer of these enzymes, depending on the enzyme and the time of binding (pro or activated form) (Mazzoni et al., 2012).

## MMPs in non-carious dentin

MMPs are believed to play an important role in the matrix remodeling that takes place during dentinogenesis. The main MMPs identified in pulp, odontoblasts and in the predentin/dentin compartments are the collagenase MMP-8, the gelatinases MMP-2 and MMP-9, stromelysin-1 (MMP-3 or proteoglycanase), the MMP-2 activator MMP-14 (MT1-MMP), MMP-13, and enamelysin (MMP-20) (Palosaari et al., 2002; Sulkala et al., 2002, 2004, 2007; Goldberg et al., 2003b; Bourd-Boittin et al., 2004; Boukpessi et al., 2008; Mazzoni et al., 2009, 2011). TIMPs, their endogenous inhibitors were also detected but at a lower level of expression (Goldberg et al., 2003b; Palosaari et al., 2003). The expression of MMP-2, the predominant MMP in sound dentin, was shown to increase gradually from the onset of dentinogenesis to reach maximal expression at day 6–7 post-natal (Bourd-Boittin et al., 2005). MMP-2 is thought to play a key role in basement membrane degradation which allows a direct epithelio-mesenchymal contact, a prerequisite for odontoblasts and ameloblasts terminal cytodifferentiation (Sahlberg et al., 1992; Heikinheimo and Salo, 1995; Sahlberg et al., 1999). At the more advanced stages of dentinogenesis, MMP-2 and MMP-9 were shown to be located near the dentino-enamel junction (Goldberg et al., 2002, 2003b), and a strong gelatinase activity was detected by in situ zymography along the mantle dentin (Pessoa et al., 2013). MMP-2 was shown to cooperate with MMP-20 in the processing of dental ECM components, and particularly in that of DSPP (Bourd-Boittin et al., 2004; Yamakoshi et al., 2006), or in the self-processing mechanism of DSPP (Godovikova and Ritchie, 2007). Importantly, MMP-2 was isolated from mature human mineralized dentin matrix (Martin-De Las Heras et al., 2000) and zymographically identified in demineralized dentin (Van Strijp et al., 2003; Mazzoni et al., 2007), suggesting a potential role in dentin ECM degradation during the carious process (Tjaderhane et al., 1998; Chaussain-Miller et al., 2006). Recent studies described a differential profile of localization and activity of the gelatinases in the different layers of human sound dentin (Boushell et al., 2011; Niu et al., 2011). High levels of MMP-2 were observed in odontoblasts where it was co-localized with TIMP-2. It was also observed in the deep dentine and at the dentinoenamel junction (Goldberg et al., 2003b). MMP-9, which colocalized with TIMP-1, was also shown to decrease from the deep to the superficial dentine layer (Niu et al., 2011). This gelatinase gradient may determine the rate of collagen degradation in pathological conditions, depending on the depth of the affected dentine.

Stromelysin-1 (MMP-3) has been identified in predentin, where it was proposed to participate in the mineralization process by degrading CS/DS (PG) (Hall et al., 1999) and in dentin where it was shown localized within the intertubular dentine, along the collagen fibrils (Mazzoni et al., 2011). Our own studies have shown that this enzyme is present in demineralized dentin in its active form, implying that it has the potential to degrade and disorganize the dentin matrix. Indeed, we have shown that when active, MMP-3 is able to cleave from the collagen scaffold several matrix proteins such as decorin, biglycan as well as four members of the SIBLING family: DSP, matrix extra-cellular phosphoglycoprotein (MEPE), bone sialoprotein (BSP) and osteopontin (OPN) (Boukpessi et al., 2008). The release by MMP-3 of (PG), such as decorin, could result in a subsequent release of the sequestered cytokines which in turn may activate other MMPs, thus potentiating the degradation of the demineralized matrix (Imai et al., 1997; Suppa et al., 2006).

## Potential mechanism(s) of MMP activation in carious dentin

MMPs are secreted to the ECM as inactive proenzymes and then require activation in order to be able to degrade matrix components. During the caries process, the acidic environment created by the release of bacterial acids can favor the activation of endogenous MMPs. Low pH was suggested to cause a conformational change within the propeptide domain of the enzyme that facilitates the cysteine switch, a critical step in the activation process (Tjaderhane et al., 1998). However, although the activated MMPs are stable in acidic pH, they can only be functional in neutral pH. Neutralization of the acids can be achieved by the dentinal buffering mechanisms (Camps and Pashley, 2000; Haapasalo et al., 2007) through the salivary buffer systems, thus allowing the pH-activated MMPs to cleave matrix components (Tjaderhane et al., 1998). In addition, the phosphorylated proteins released from the collagen scaffold by bacterial acids could interact with TIMP-inhibited MMPs within the carious lesion and re-activate them, enhancing the degradation process (Fedarko et al., 2004). Another enzyme family, the cysteine cathepsins, was identified in dentin (Tersariol et al., 2010) and in saliva of patients with periodontal conditions. They have also been reported in human carious dentin (Van Strijp et al., 2003) and it was suggested that these enzymes, when active, can in turn activate latent MMPs (Van Strijp et al., 2003). Along this line, a recent study reported a stronger immunostaining of cathepsins B in carious dentin, observed in dentin tubules and in odontoblasts, when compared with sound dentin (Nascimento et al., 2011). In addition, carious dentin displayed increased cysteine cathepsin activity, which was even greater in deep lesions with pulp exposure. Interestingly, it was also shown in this study that MMP activity in the saliva was higher in patients with active compared to chronic carious lesions, while salivary cathepsins did not show respective correlation. The reason may be that saliva is rich in cystatins (Dickinson, 2002), potent cysteine peptidase inhibitors. On the other hand, cathepsin activity increases with increasing depth of the lesion, indicating the role of pulp tissue-derived enzymes (Nascimento et al., 2011). Taken together, these studies suggest that cysteine cathepsins may participate in the carious process at least partly by activating latent MMPs.

## Dentin matrix degradation during the carious process

**Figure 1:**
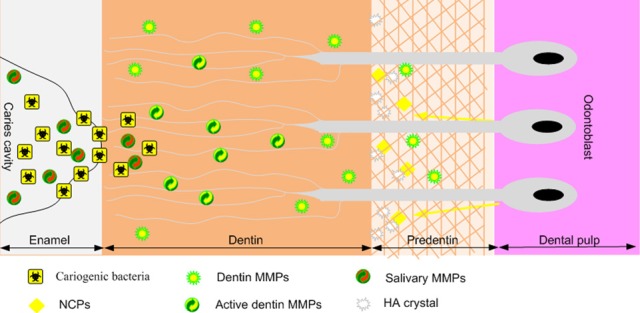
**Schematic representation of MMP activity during the dentin carious process.** Cariogenic bacteria present in the caries cavity release acids such as lactic acid that reduce the local pH. The resulting acidic environment demineralizes the dentin matrix and induces the activation of host MMPs derived from dentin or saliva (which bathes the caries cavity). Once the local pH is neutralized by salivary buffer systems, activated MMPs degrade the demineralized dentin matrix.

**Figure 2:**
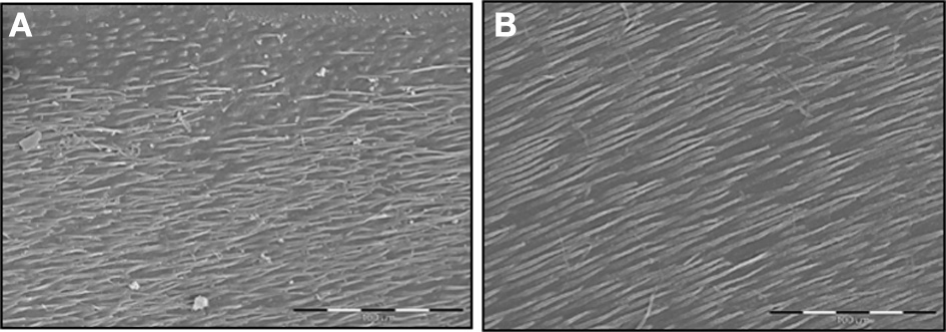
**SEM examination of the effects of MMP-2 on the dentin matrix through low viscosity resin replicas. (A)** Standardized human dentin cubes were treated for 24 h with 0.5 M lactic (pH 4.8) and resin penetration in demineralized dentin was observed by resin replicas. The low viscosity resin penetrates in the main dentin tubules. **(B)** The treatment of demineralized dentin cubes by activated recombinant MMP-2 shows a deeper resin penetration into the main dentin tubules, suggesting increased dentin matrix degradation. Bar = 100 mm.

Host MMPs derived from dentin or saliva have been shown to be able to degrade the dentin matrix which has been previously demineralized by bacterial acids (Tjaderhane et al., 1999; Chaussain-Miller et al., 2006) (Figure. 1). Saliva contains several MMPs including collagenases and gelatinases derived from either the gingival crevicular fluid or the secretion of salivary glands, MMP-9 being the most abundant as it is derived from both sources (Van Strijp et al., 2003). Indeed, the incubation of demineralized dentin slabs with acid-pretreated saliva resulted in the degradation of the organic matrix (Van Strijp et al., 2003). As saliva bathes the carious lesions, it is not surprising that the active form of MMP-9 was systematically detected by zymography performed on dentin extracts from several carious teeth (Tjaderhane et al., 1998). Together these studies indicate that salivary MMPs may have a strong contribution to dentin matrix degradation during the caries process. Carious lesions were also found to contain both latent and active forms of MMP-3, MMP-2 and MMP-8 (Tjaderhane et al., 1998; Mazzoni et al., 2007; Sulkala et al., 2007; Boukpessi et al., 2008). Along this line, dentin protein extracts obtained from the different dentin layers of decayed teeth were shown to have their gelatinase activity gradually increased from healthy dentin extracts to soft infected dentin extracts (superficial soft carious lesion, inner soft carious lesion, affected dentin, sound dentin) (Charadram et al., 2012). These observations confirm that endogenous MMP-2 contained within the sound dentin is activated during the carious process (Figure. 2). It has also been suggested by immunohistochemical observations that the endogenous MMP-2 level may be increased through the induction of MMP-2 synthesis by the presence of caries (Toledano et al., 2010). This was supported by a study showing that MMP-2 and TIMP-2 gene expressions were significantly up-regulated in odontoblasts adjacent to the carious lesion (Charadram et al., 2012). Interestingly, several studies have reported that in the cancer context, MMP expression by tumor cells was up-regulated by an acidic extracellular pH (Kato et al., 2005; Rofstad et al., 2006). This can suggest that acidic pH during the carious process may both induce MMP expression by the odontoblasts and favor their activation, potentiating MMP proteolytic capacity. In addition, the expression of MT1-MMP, a potent activator of MMP-2 and MMP-20 (Palosaari et al., 2002), was also dramatically increased in these cells, enhancing dentin matrix degradation. In addition, MMP-2, MMP- 20 and cathepsin B were shown to be present in dentinal fluid, where they may contribute to peritubular dentin degradation especially in young patients who have large and numerous dentin tubules (Sulkala et al., 2002; Boushell et al., 2008; Tersariol et al., 2010). Importantly, a study performed in a rat caries model has shown that MMP inhibition by several synthetic inhibitors reduced dentin caries progression under fissures (Sulkala et al., 2001). This study not only confirms the role of host MMPs in the caries process but also raises the possibility of using MMP inhibitors for impairing dentin matrix degradation during the caries.

## MMP inhibitors for the treatment of carious dentin

Considering the potential role of MMPs in dentin ECM degradation, it seems logical that, when associated with a thorough control of the caries (Selwitz et al., 2007), MMP inhibition would help to control dentin caries progression. This notion was supported by studies in rat caries models where dentin caries progression was delayed by intra-oral administration of chemical MMP inhibitors (Tjaderhane et al., 1999; Sulkala et al., 2001). Several effective MMP inhibitors have been described. Tetracyclines and their derivatives, doxicycline and minocycline are able to inhibit MMP activity independent of their antimicrobial action. They are commonly used as antibiotics in the treatment of periodontitis (Ryan et al., 1996) and have been shown both *in vitro* and *in vivo* to inhibit MMP-1, MMP-2, MMP-8 and MMP-12 (Golub et al., 1995; Lauhio et al., 1995). Another effective and safe MMP inhibitor is the non-antimicrobial chemically modified tetracyclines (CMTs), which can inhibit both the release and the activity of MMPs (Golub et al., 1998; Ramamurthy et al., 1998). Zoledronate is a third generation bisphosphonate which has the ability to inhibit MMP proteolytic activities (Teronen et al., 1997; Boissier et al., 2000). Sulkala et al. (2001) have shown that the systemic MMP inhibition with CMT-3 and zoledronate *in vivo* suppressed the progression of dental caries under fissures, indicating that systemic administration of MMPs inhibitors could be effective in caries prevention (Sulkala et al., 2001). Indeed, the authors observed a reduction in the progression of caries in rats treated with these MMP inhibitors, though no synergistic potentiating effect of these two compounds could been demonstrated.

In humans, MMP inhibitors would preferably be administered locally to treat dental caries by either incorporating them in topical preparations for daily use or by applying them directly on the dentin surface, depending on the clinical situation. When treating coronal caries, especially in young patients with deep and active caries lesions, a solution containing MMP inhibitors may be applied directly to the lesion after the mechanical removal of the caries and before restoration. A second strategy would be to incorporate these inhibitors in mouth rinses or toothpastes to prevent root caries progression. Several synthetic MMP inhibitors are already used in the dental practice. The MMP inhibitory action of most of them is based on their zinc-/calcium-chelating groups, since MMPs require metal ions (calcium and zinc) for their catalytic activity (Gendron et al., 1999). Among them, Ethylenediaminetetraacetic acid (EDTA), which is an effective zinc and calcium chelator, was recently shown to inhibit the degradation by acid-activated endogenous MMPs of dentin beams treated for 1 min with 17% EDTA (Thompson et al., 2012). Chlorhexidine digluconate (CHX) has also potent MMP inhibitor effects that also involve a calcium-chelating mechanism (Gendron et al., 1999). These inhibitors have been shown to improve the integrity of the hybrid layers obtained by a simplified etch-and-rinse adhesive after dentin caries removal (Carrilho et al., 2007). The local application of 2% CHX for 1 min to the etched dentin surface just before applying the dentin bonding primer was able to inhibit the degradation of the hybrid layer by MMPs for at least 14 months. This clinical study highlights that CHX, a MMP inhibitor already used in dental practice, is able to impair dentin matrix degradation. Interestingly, Scaffa et al. demonstrated that CHX was also a potent inhibitor of the cysteine cathepsin enzymes (Scaffa et al., 2012), which were shown to be present and active in sound and carious dentin (Tersariol et al., 2010; Nascimento et al., 2011).

A different group of MMP inhibitors include those derived from natural sources. Green tea polyphenols, especially epigallocatechin gallate (EGCG), were found to have potent and distinct inhibitory activity against MT1-MMP, resulting in the decrease of MMP-2 activation. Furthermore, EGCG inhibits directly MMP-2 and MMP-9 (Demeule et al., 2000; Garbisa et al., 2001; Dell'aica et al., 2002), and was recently shown to inhibit dentinal erosion, along with other known MMP inhibitors (Kato et al., 2010). Grape seed extract (GSE) has been shown to suppress lipopolysaccharide-induced MMP secretion by macrophages and to inhibit MMP-1 and MMP-9 activity in periodontitis (La et al., 2009a). Recent *in vitro* studies demonstrated that GSE inhibited the demineralization and/or promoted the remineralization of artificial root carious lesions under dynamic pH- cycling conditions (Xie et al., 2008; Pavan et al., 2011). The MMP-inhibitory effects of these or other natural substances such as cranberry proanthocyanidins (La et al., 2009b) suggest that they could be effective in slowing down dentin caries progression. The fact that these molecules are devoid of undesirable side effects, when compared with those of synthetic drugs, makes them particularly attractive for the treatment of dentin caries, since they can be safely applied directly on the affected tooth or integrated in daily-used topical products.

## Generation of bioactive peptides from dentin matrix degradation

By cleaving large insoluble ECM components, MMPs are known to liberate bioactive fragments and cytokines (Mott and Werb, 2004). The release of active peptides by the enzymatic cleavage of dentin matrix proteins by MMPs during the carious process was also suggested (Chaussain-Miller et al., 2006). These peptides, which often retain the activity of the parent protein (Dean and Overall, 2007), or display a function different from those of the protein they are derived from, can affect several biological properties in terms of proliferation, angiogenesis, differentiation that may favor the pulp healing process (Dean et al., 2007; Smith et al., 2012a). In particular, the degradation of matrix (PG), which often bind and sequester growth factors, by proteases such as the MMP-3 would cause the release and activation of these factors within the carious lesion and can as a result impact pulp healing (Smith et al., 2012b). The gelatinase MMP-2 has been proposed to be able to release active TGF-β 2 in the carious lesion, which can then stimulate diverse repair processes (Charadram et al., 2012).

**Figure 3:**
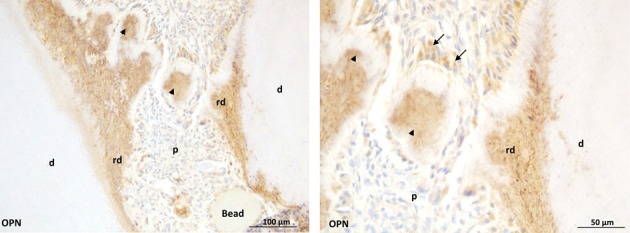
**Early dentin repair in an injured rat pulp model induced by the implantation of C-DMP1 peptide mimicking an MMP2 cleaved peptide of DMP1.Beads soaked with c-DMP1 polypeptide were implanted in the injured pulp of young rats.** At day 7, osteopontin (OPN) immunohistochemistry shows strong immunoreactivity of reparative structures (arrowheads) observed in the injured pulp resembling mineralization foci (Mckee et al., 1993). Bridge formation at longer time points may partly result from growth and spreading of these mineralization foci. At higher magnification (right panel), immunopositive cells (arrows) are observed adjacent to the reparative structures.p: pulp; rd: reactionary dentin; d: dentin.

Using an injured rat pulp model to test the biological properties of such cleavage products derived from the (ECM), we demonstrated that a polypeptide containing the C-terminal part of DMP1, and mimicking DMP1 cleavage product by MMP-2, was able to enhance pulp healing capacity when implanted in a rat pulp-injury model (Chaussain et al., 2009). A dense and continuous reparative dentin bridge was rapidly observed after implantation and the cells boarding the bridge presented a characteristic polarized odontoblastic phenotype, organized as a palisade and expressing the odontoblastic markers DSP and DMP1 (Figure 3). The use of such ECM-derived bioactive peptides associated with restorative materials constitutes a promising therapeutical option for tooth repair (Goldberg et al., 2008). In addition, these peptides may also be used for bone regeneration, potentially associated with engineered scaffolds and mesenchymal stem cells (Mao et al., 2006), as dentin and bone are similar in several aspects, especially pertaining to the composition of the ECM (Opsahl Vital et al., 2012).

In conclusion, accumulating data have demonstrated that endogenous enzymes contained in dentin and saliva are involved in the dentin caries process. These findings are important, since they open new options for caries prevention and treatment. Slowing down or preventing the irreversible destruction of the organic matrix would allow for natural healing of the lesion by remineralization. MMP inhibitors may prove to be useful in the prevention of dentin caries progression. However, the outcomes of this approach will depend on the management of the caries risk, involving the control of caries risk factors such as cariogenic biofilm, diet, inadequate salivary flow rate and insufficient fluoride exposure (Selwitz et al., 2007).

### Conflict of interest statement

The authors declare that the research was conducted in the absence of any commercial or financial relationships that could be construed as a potential conflict of interest.

## References

[B1] Addison W. N. Mckee M. D. (2010). ASARM mineralization hypothesis: a bridge to progress. J. Bone Miner. Res. 25, 1191–1192 10.1002/jbmr.110 20496373

[B2] Boissier S. Ferreras M. Peyruchaud O. Magnetto S. Ebetino F. H. Colombel M. (2000). Bisphosphonates inhibit breast and prostate carcinoma cell invasion, an early event in the formation of bone metastases. Cancer Res. 60, 2949–2954 10850442

[B3] Boukpessi T. Menashi S. Camoin L. Tencate J. M. Goldberg M. Chaussain-Miller C. (2008). The effect of stromelysin-1 (MMP-3) on non-collagenous extracellular matrix proteins of demineralized dentin and the adhesive properties of restorative resins. Biomaterials 29, 4367–4373 10.1016/j.biomaterials.2008.07.035 18760468

[B4] Bourd-Boittin K. Fridman R. Fanchon S. Septier D. Goldberg M. Menashi S. (2005). Matrix metalloproteinase inhibition impairs the processing, formation and mineralization of dental tissues during mouse molar development. Exp. Cell Res. 304, 493–505 10.1016/j.yexcr.2004.11.024 15748894

[B5] Bourd-Boittin K. Septier D. Hall R. Goldberg M. Menashi S. (2004). Immunolocalization of enamelysin (matrix metalloproteinase-20) in the forming rat incisor. J. Histochem. Cytochem. 52, 437–445 10.1177/002215540405200402 15033995

[B6] Boushell L. W. Kaku M. Mochida Y. Bagnell R. Yamauchi M. (2008). Immunohistochemical localization of matrixmetalloproteinase-2 in human coronal dentin. Arch. Oral Biol. 53, 109–116 10.1016/j.archoralbio.2007.09.012 18001692 PMC2258007

[B7] Boushell L. W. Kaku M. Mochida Y. Yamauchi M. (2011). Distribution and relative activity of matrix metalloproteinase-2 in human coronal dentin. Int. J. Oral Sci. 3, 192–199 10.4248/IJOS11070 22010577 PMC3469976

[B8] Brinckerhoff C. E. Matrisian L. M. (2002). Matrix metalloproteinases: a tail of a frog that became a prince. Nat. Rev. Mol. Cell Biol. 3, 207–214 10.1038/nrm763 11994741

[B9] Bronckers A. L. Price P. A. Schrijvers A. Bervoets T. J. Karsenty G. (1998). Studies of osteocalcin function in dentin formation in rodent teeth. Eur. J. Oral Sci. 106, 795–807 10.1046/j.0909-8836.1998.eos106306.x 9672102

[B10] Camps J. Pashley D. H. (2000). Buffering action of human dentin *in vitro*. J. Adhes. Dent. 2, 39–50 11317407

[B11] Carrilho M. R. Carvalho R. M. De Goes M. F. Di Hipolito V. Geraldeli S. Tay F. R. (2007). Chlorhexidine preserves dentin bond *in vitro*. J. Dent. Res. 86, 90–94 10.1177/154405910708600115 17189470 PMC2248723

[B12] Charadram N. Farahani R. M. Harty D. Rathsam C. Swain M. V. Hunter N. (2012). Regulation of reactionary dentin formation by odontoblasts in response to polymicrobial invasion of dentin matrix. Bone 50, 265–275 10.1016/j.bone.2011.10.031 22079283 PMC3246533

[B13] Chaussain C. Eapen A. S. Huet E. Floris C. Ravindran S. Hao J. (2009). MMP2-cleavage of DMP1 generates a bioactive peptide promoting differentiation of dental pulp stem/progenitor cell. Eur. Cell. Mater. 18, 84–95 19908197 10.22203/ecm.v018a08 PMC3092783

[B14] Chaussain-Miller C. Fioretti F. Goldberg M. Menashi S. (2006). The role of matrix metalloproteinases (MMPs) in human caries. J. Dent. Res. 85, 22–32 10.1177/154405910608500104 16373676

[B15] David V. Quarles L. D. (2010). ASARM mineralization hypothesis: a bridge too far? J. Bone Miner. Res. 25, 692–694 10.1002/jbmr.69 20200985 PMC3153325

[B16] Dean R. A. Overall C. M. (2007). Proteomics discovery of metalloproteinase substrates in the cellular context by iTRAQ labeling reveals a diverse MMP-2 substrate degradome. Mol. Cell Proteomics 6, 611–623 10.1074/mcp.M600341-MCP200 17200105

[B17] Dean R. A. Smith D. Overall C. M. (2007). Proteomic identification of cellular protease substrates using isobaric tags for relative and absolute quantification (iTRAQ). Curr. Protoc. Protein Sci. Unit 21.18. 10.1002/0471140864.ps2118s49 18429318

[B18] Dell'aica I. Dona M. Sartor L. Pezzato E. Garbisa S. (2002). (-)Epigallocatechin-3-gallate directly inhibits MT1-MMP activity, leading to accumulation of nonactivated MMP-2 at the cell surface. Lab. Invest. 82, 1685–1693 10.1097/01.LAB.0000043122.00384.91 12480918

[B19] Demeule M. Brossard M. Page M. Gingras D. Beliveau R. (2000). Matrix metalloproteinase inhibition by green tea catechins. Biochim. Biophys. Acta 1478, 51–60 10.1016/S0167-4838(00)00009-1 10719174

[B20] Dickinson D. P. (2002). Salivary (SD-type) cystatins: over one billion years in the making–but to what purpose? Crit. Rev. Oral Biol. Med. 13, 485–508 10.1177/154411130201300606 12499242

[B21] Embery G. Hall R. Waddington R. Septier D. Goldberg M. (2001). Proteoglycans in dentinogenesis. Crit. Rev. Oral Biol. Med. 12, 331–349 10.1177/10454411010120040401 11603505

[B22] Fedarko N. S. Jain A. Karadag A. Fisher L. W. (2004). Three small integrin binding ligand N-linked glycoproteins (SIBLINGs) bind and activate specific matrix metalloproteinases. FASEB J. 18, 734–736 10.1096/fj.03-0966fje 14766790

[B23] Fisher L. W. Fedarko N. S. (2003). Six genes expressed in bones and teeth encode the current members of the SIBLING family of proteins. Connect. Tissue Res. 44(Suppl. 1), 33–40 10.1080/713713644 12952171

[B24] Garbisa S. Sartor L. Biggin S. Salvato B. Benelli R. Albini A. (2001). Tumor gelatinases and invasion inhibited by the green tea flavanol epigallocatechin-3-gallate. Cancer 91, 822–832 10.1002/1097-0142(20010215)91:4<822::AID-CNCR1070>3.0.CO;2-G 11241252

[B25] Gendron R. Grenier D. Sorsa T. Mayrand D. (1999). Inhibition of the activities of matrix metalloproteinases 2, 8, and 9 by chlorhexidine. Clin. Diagn. Lab. Immunol. 6, 437–439 10225852 10.1128/cdli.6.3.437-439.1999 PMC103739

[B26] Godovikova V. Ritchie H. H. (2007). Dynamic processing of recombinant dentin sialoprotein-phosphophoryn protein. J. Biol. Chem. 282, 31341–31348 10.1074/jbc.M702605200 17698853

[B27] Goldberg M. Smith A. J. (2004). Cells and extracellular matrices of dentin and pulp: a biological basis for repair and tissue engineering. Crit. Rev. Oral Biol. Med. 15, 13–27 10.1177/154411130401500103 14761897

[B28] Goldberg M. Lacerda-Pinheiro S. Priam F. Jegat N. Six N. Bonnefoix M. (2008). Matricellular molecules and odontoblast progenitors as tools for dentin repair and regeneration. Clin. Oral Investig. 12, 109–112 10.1007/s00784-007-0172-6 18157557 PMC2834229

[B29] Goldberg M. Rapoport O. Septier D. Palmier K. Hall R. Embery G. (2003a). Proteoglycans in predentin: the last 15 micrometers before mineralization. Connect. Tissue Res. 44(Suppl. 1), 184–188 10.1080/03008200390152304 12952195

[B30] Goldberg M. Septier D. Bourd K. Hall R. George A. Goldberg H. (2003b). Immunohistochemical localization of MMP-2, MMP-9, TIMP-1, and TIMP-2 in the forming rat incisor. Connect. Tissue Res. 44, 143–153 10.1080/03008200390223927 14504034

[B31] Goldberg M. Septier D. Escaig-Haye F. (1987). Glycoconjugates in dentinogenesis and dentine. Prog. Histochem. Cytochem. 17, 1–112 10.1016/S0079-6336(87)80001-3 3575752

[B32] Goldberg M. Septier D. Bourd K. Hall R. Jeanny J. C. Jonet L. (2002). The dentino-enamel junction revisited. Connect. Tissue Res. 43, 482–489 10.1080/03008200290000817 12489202

[B33] Golub L. M. Lee H. M. Ryan M. E. Giannobile W. V. Payne J. Sorsa T. (1998). Tetracyclines inhibit connective tissue breakdown by multiple non-antimicrobial mechanisms. Adv. Dent. Res. 12, 12–26 10.1177/08959374980120010501 9972117

[B34] Golub L. M. Sorsa T. Lee H. M. Ciancio S. Sorbi D. Ramamurthy N. S. (1995). Doxycycline inhibits neutrophil (PMN)-type matrix metalloproteinases in human adult periodontitis gingiva. J. Clin. Periodontol. 22, 100–109 10.1111/j.1600-051X.1995.tb00120.x 7775665

[B35] Haapasalo M. Qian W. Portenier I. Waltimo T. (2007). Effects of dentin on the antimicrobial properties of endodontic medicaments. J. Endod. 33, 917–925 10.1016/j.joen.2007.04.008 17878075

[B36] Hall R. Septier D. Embery G. Goldberg M. (1999). Stromelysin-1 (MMP-3) in forming enamel and predentine in rat incisor-coordinated distribution with proteoglycans suggests a functional role. Histochem. J. 31, 761–770 10.1023/A:1003945902473 10661319

[B37] Heikinheimo K. Salo T. (1995). Expression of basement membrane type IV collagen and type IV collagenases (MMP-2 and MMP-9) in human fetal teeth. J. Dent. Res. 74, 1226–1234 10.1177/00220345950740051301 7790601

[B38] Imai K. Hiramatsu A. Fukushima D. Pierschbacher M. D. Okada Y. (1997). Degradation of decorin by matrix metalloproteinases: identification of the cleavage sites, kinetic analyses and transforming growth factor-beta1 release. Biochem. J. 322(Pt 3), 809–814 9148753 10.1042/bj3220809 PMC1218259

[B39] Kaipatur N. R. Murshed M. Mckee M. D. (2008). Matrix Gla protein inhibition of tooth mineralization. J. Dent. Res. 87, 839–844 10.1177/154405910808700907 18719210

[B40] Kato M. T. Leite A. L. Hannas A. R. Buzalaf M. A. (2010). Gels containing MMP inhibitors prevent dental erosion *in situ*. J. Dent. Res. 89, 468–472 10.1177/0022034510363248 20200409

[B41] Kato Y. Lambert C. A. Colige A. C. Mineur P. Noel A. Frankenne F. (2005). Acidic extracellular pH induces matrix metalloproteinase-9 expression in mouse metastatic melanoma cells through the phospholipase D-mitogen-activated protein kinase signaling. J. Biol. Chem. 280, 10938–10944 10.1074/jbc.M411313200 15657063

[B42] Kawasaki K. Featherstone J. D. (1997). Effects of collagenase on root demineralization. J. Dent. Res. 76, 588–595 10.1177/00220345970760011001 9042082

[B43] La V. D. Bergeron C. Gafner S. Grenier D. (2009a). Grape seed extract suppresses lipopolysaccharide-induced matrix metalloproteinase (MMP) secretion by macrophages and inhibits human MMP-1 and -9 activities. J. Periodontol. 80, 1875–1882 10.1902/jop.2009.090251 19905958

[B44] La V. D. Howell A. B. Grenier D. (2009b). Cranberry proanthocyanidins inhibit MMP production and activity. J. Dent. Res. 88, 627–632 10.1177/0022034509339487 19641150

[B45] Lauhio A. Salo T. Tjaderhane L. Lahdevirta J. Golub L. M. Sorsa T. (1995). Tetracyclines in treatment of rheumatoid arthritis. Lancet 346, 645–646 10.1016/S0140-6736(95)91484-6 7651040

[B46] Mao J. J. Giannobile W. V. Helms J. A. Hollister S. J. Krebsbach P. H. Longaker M. T. (2006). Craniofacial tissue engineering by stem cells. J. Dent. Res. 85, 966–979 10.1177/154405910608501101 17062735 PMC2571078

[B47] Martin-De Las Heras S. Valenzuela A. Overall C. M. (2000). Gelatinase A in human dentin as a new biochemical marker for age estimation. J. Forensic Sci. 45, 807–811 10914574

[B47a] Mazzoni A. Breschi L. Carrilho M. Nascimento F. D. Orsini G. Ruggeri A. (2012). A review on nature, role and functions of dentin non-collagenous proteins. Part II: enzymes, serum proteins and growth factors. Endod. Top. 21, 19–40 10.1111/j.1601-1546.2012.00268.x

[B48] Mazzoni A. Mannello F. Tay F. R. Tonti G. A. Papa S. Mazzotti G. (2007). Zymographic analysis and characterization of MMP-2 and -9 forms in human sound dentin. J. Dent. Res. 86, 436–440 10.1177/154405910708600509 17452564

[B50] Mazzoni A. Papa V. Nato F. Carrilho M. Tjaderhane L. Ruggeri A. (2011). Immunohistochemical and biochemical assay of MMP-3 in human dentine. J. Dent. 39, 231–237 10.1016/j.jdent.2011.01.001 21215789 PMC3815524

[B51] Mazzoni A. Pashley D. H. Tay F. R. Gobbi P. Orsini G. Ruggeri A. (2009). Immunohistochemical identification of MMP-2 and MMP-9 in human dentin: correlative FEI-SEM/TEM analysis. J. Biomed. Mater. Res. A 88A, 697–703 10.1002/jbm.a.31920 18335530

[B52] Mckee M. D. Farach-Carson M. C. Butler W. T. Hauschka P. V. Nanci A. (1993). Ultrastructural immunolocalization of noncollagenous (osteopontin and osteocalcin) and plasma (albumin and alpha 2HS-glycoprotein) proteins in rat bone. J. Bone Miner. Res. 8, 485–496 10.1002/jbmr.5650080413 8475798

[B53] Mott J. D. Werb Z. (2004). Regulation of matrix biology by matrix metalloproteinases. Curr. Opin. Cell Biol. 16, 558–564 10.1016/j.ceb.2004.07.010 15363807 PMC2775446

[B54] Nascimento F. D. Minciotti C. L. Geraldeli S. Carrilho M. R. Pashley D. H. Tay F. R. (2011). Cysteine cathepsins in human carious dentin. J. Dent. Res. 90, 506–511 10.1177/0022034510391906 21248362 PMC3144127

[B55] Niu L. N. Zhang L. Jiao K. Li F. Ding Y. X. Wang D. Y. (2011). Localization of MMP-2, MMP-9, TIMP-1, and TIMP-2 in human coronal dentine. J. Dent. 39, 536–542 10.1016/j.jdent.2011.05.004 21641958

[B56] Onishi T. Ogawa T. Hayashibara T. Hoshino T. Okawa R. Ooshima T. (2005). Hyper-expression of osteocalcin mRNA in odontoblasts of Hyp mice. J. Dent. Res. 84, 84–88 10.1177/154405910508400115 15615882

[B57] Opsahl Vital S. Gaucher C. Bardet C. Rowe P. S. George A. Linglart A. (2012). Tooth dentin defects reflect genetic disorders affecting bone mineralization. Bone 50, 989–997 10.1016/j.bone.2012.01.010 22296718 PMC3345892

[B58] Palosaari H. Ding Y. Larmas M. Sorsa T. Bartlett J. D. Salo T. (2002). Regulation and interactions of MT1-MMP and MMP-20 in human odontoblasts and pulp tissue *in vitro*. J. Dent. Res. 81, 354–359 10.1177/154405910208100513 12097451

[B59] Palosaari H. Pennington C. J. Larmas M. Edwards D. R. Tjaderhane L. Salo T. (2003). Expression profile of matrix metalloproteinases (MMPs) and tissue inhibitors of MMPs in mature human odontoblasts and pulp tissue. Eur. J. Oral Sci. 111, 117–127 10.1034/j.1600-0722.2003.00026.x 12648263

[B60] Pavan S. Xie Q. Hara A. T. Bedran-Russo A. K. (2011). Biomimetic approach for root caries prevention using a proanthocyanidin-rich agent. Caries Res. 45, 443–447 10.1159/000330599 21860242 PMC3169369

[B61] Pessoa J. I. Guimaraes G. N. Viola N. V. Da Silva W. J. De Souza A. P. Tjaderhane L. (2013). *In situ* study of the gelatinase activity in demineralized dentin from rat molar teeth. Acta Histochem. 115, 245–251 10.1016/j.acthis.2012.07.002 22897943

[B62] Qin C. Baba O. Butler W. T. (2004). Post-translational modifications of sibling proteins and their roles in osteogenesis and dentinogenesis. Crit. Rev. Oral Biol. Med. 15, 126–136 10.1177/154411130401500302 15187031

[B63] Ramamurthy N. S. Schroeder K. L. Mcnamara T. F. Gwinnett A. J. Evans R. T. Bosko C. (1998). Root-surface caries in rats and humans: inhibition by a non-antimicrobial property of tetracyclines. Adv. Dent. Res. 12, 43–50 10.1177/08959374980120011801 9972121

[B64] Rofstad E. K. Mathiesen B. Kindem K. Galappathi K. (2006). Acidic extracellular pH promotes experimental metastasis of human melanoma cells in athymic nude mice. Cancer Res. 66, 6699–6707 10.1158/0008-5472.CAN-06-0983 16818644

[B65] Rowe P. S. (2012). The chicken or the egg: PHEX, FGF23 and SIBLINGs unscrambled. Cell Biochem. Funct. 30, 355–375 10.1002/cbf.2841 22573484 PMC3389266

[B66] Rowe P. S. De Zoysa P. A. Dong R. Wang H. R. White K. E. Econs M. J. (2000). MEPE, a new gene expressed in bone marrow and tumors causing osteomalacia. Genomics 67, 54–68 10.1006/geno.2000.6235 10945470

[B67] Ryan M. E. Ramamurthy S. Golub L. M. (1996). Matrix metalloproteinases and their inhibition in periodontal treatment. Curr. Opin. Periodontol. 3, 85–96 8624573

[B68] Sahlberg C. Reponen P. Tryggvason K. Thesleff I. (1992). Association between the expression of murine 72kDa type IV collagenase by odontoblasts and basement membrane degradation during mouse tooth development. Arch. Oral Biol. 37, 1021–1030 10.1016/0003-9969(92)90034-6 1471951

[B69] Sahlberg C. Reponen P. Tryggvason K. Thesleff I. (1999). Timp-1, -2 and -3 show coexpression with gelatinases A and B during mouse tooth morphogenesis. Eur. J. Oral Sci. 107, 121–130 10.1046/j.0909-8836.1999.eos107208.x 10232461

[B70] Scaffa P. M. Vidal C. M. Barros N. Gesteira T. F. Carmona A. K. Breschi L. (2012). Chlorhexidine inhibits the activity of dental cysteine cathepsins. J. Dent. Res. 91, 420–425 10.1177/0022034511435329 22266526

[B71] Selwitz R. H. Ismail A. I. Pitts N. B. (2007). Dental caries. Lancet 369, 51–59 10.1016/S0140-6736(07)60031-2 17208642

[B72] Smith A. J. Scheven B. A. Takahashi Y. Ferracane J. L. Shelton R. M. Cooper P. R. (2012a). Dentine as a bioactive extracellular matrix. Arch. Oral Biol. 57, 109–121 10.1016/j.archoralbio.2011.07.008 21855856

[B73] Smith J. G. Smith A. J. Shelton R. M. Cooper P. R. (2012b). Recruitment of dental pulp cells by dentine and pulp extracellular matrix components. Exp. Cell Res. 318, 2397–2406 10.1016/j.yexcr.2012.07.008 22819733

[B74] Sulkala M. Larmas M. Sorsa T. Salo T. Tjaderhane L. (2002). The localization of matrix metalloproteinase-20 (MMP-20, enamelysin) in mature human teeth. J. Dent. Res. 81, 603–607 10.1177/154405910208100905 12202640

[B75] Sulkala M. Paakkonen V. Larmas M. Salo T. Tjaderhane L. (2004). Matrix metalloproteinase-13 (MMP-13, collagenase-3) is highly expressed in human tooth pulp. Connect. Tissue Res. 45, 231–237 10.1080/03008200490885788 15763932

[B76] Sulkala M. Tervahartiala T. Sorsa T. Larmas M. Salo T. Tjaderhane L. (2007). Matrix metalloproteinase-8 (MMP-8) is the major collagenase in human dentin. Arch. Oral Biol. 52, 121–127 10.1016/j.archoralbio.2006.08.009 17045563

[B77] Sulkala M. Wahlgren J. Larmas M. Sorsa T. Teronen O. Salo T. (2001). The effects of MMP inhibitors on human salivary MMP activity and caries progression in rats. J. Dent. Res. 80, 1545–1549 10.1177/00220345010800061301 11499510

[B78] Suppa P. Ruggeri A. Jr. Tay F. R. Prati C. Biasotto M. (2006). Reduced antigenicity of type I collagen and proteoglycans in sclerotic dentin. J. Dent. Res. 85, 133–137 10.1177/154405910608500204 16434730 PMC2245799

[B79] Teronen O. Konttinen Y. T. Lindqvist C. Salo T. Ingman T. Lauhio A. (1997). Inhibition of matrix metalloproteinase-1 by dichloromethylene bisphosphonate (clodronate). Calcif. Tissue Int. 61, 59–61 10.1007/s002239900295 9192515

[B80] Tersariol I. L. Geraldeli S. Minciotti C. L. Nascimento F. D. Paakkonen V. Martins M. T. (2010). Cysteine cathepsins in human dentin-pulp complex. J. Endod. 36, 475–481 10.1016/j.joen.2009.12.034 20171366

[B81] Thompson J. M. Agee K. Sidow S. J. Mcnally K. Lindsey K. Borke J. (2012). Inhibition of endogenous dentin matrix metalloproteinases by ethylenediaminetetraacetic acid. J. Endod. 38, 62–65 10.1016/j.joen.2011.09.005 22152622 PMC3240849

[B82] Tjaderhane L. Larjava H. Sorsa T. Uitto V. J. Larmas M. Salo T. (1998). The activation and function of host matrix metalloproteinases in dentin matrix breakdown in caries lesions. J. Dent. Res. 77, 1622–1629 10.1177/00220345980770081001 9719036

[B83] Tjaderhane L. Sulkala M. Sorsa T. Teronen O. Larmas M. Salo T. (1999). The effect of MMP inhibitor metastat on fissure caries progression in rats. Ann. N. Y. Acad. Sci. 878, 686–688 10.1111/j.1749-6632.1999.tb07762.x 10415808

[B84] Toledano M. Nieto-Aguilar R. Osorio R. Campos A. Osorio E. Tay F. R. (2010). Differential expression of matrix metalloproteinase-2 in human coronal and radicular sound and carious dentine. J. Dent. 38, 635–640 10.1016/j.jdent.2010.05.001 20452393

[B85] Van Strijp A. J. Jansen D. C. Degroot J. Ten Cate J. M. Everts V. (2003). Host-derived proteinases and degradation of dentine collagen in situ. Caries Res. 37, 58–65 10.1159/000068223 12566641

[B86] Visse R. Nagase H. (2003). Matrix metalloproteinases and tissue inhibitors of metalloproteinases: structure, function, and biochemistry. Circ. Res. 92, 827–839 10.1161/01.RES.0000070112.80711.3D 12730128

[B87] Xie Q. Bedran-Russo A. K. Wu C. D. (2008). *In vitro* remineralization effects of grape seed extract on artificial root caries. J. Dent. 36, 900–906 10.1016/j.jdent.2008.07.011 18819742 PMC2583354

[B88] Yamakoshi Y. Hu J. C. Iwata T. Kobayashi K. Fukae M. Simmer J. P. (2006). Dentin sialophosphoprotein is processed by MMP-2 and MMP-20 *in vitro* and *in vivo*. J. Biol. Chem. 281, 38235–38243 10.1074/jbc.M607767200 17046814

